# Thermo-Mechanical Reliability Study of Through Glass Vias in 3D Interconnection

**DOI:** 10.3390/mi13101799

**Published:** 2022-10-21

**Authors:** Jin Zhao, Zuohuan Chen, Fei Qin, Daquan Yu

**Affiliations:** 1 Faculty of Materials and Manufacturing, Beijing University of Technology, Beijing 100124, China; 2 School of Electronic Science and Engineering, Xiamen University, Xiamen 361005, China

**Keywords:** thermo-mechanical reliability, through glass vias, finite-element method, 3D vertical interconnection, crack

## Abstract

Three-dimensional (3D) interconnection technology based on glass through vias (TGVs) has been used to integrate passive devices, and optoelectronic devices due to its superior electrical qualities, outstanding mechanical stability, and lower cost. Nevertheless, the performance and reliability of the device will be impacted by the thermal stress brought on by the mismatch of the coefficient of thermal expansion among multi-material structures and the complicated structure of TGV. This paper focuses on thermal stress evolution in different geometric and material parameters and the development of a controlled method for filling polymers in TGV interconnected structures. In addition, a numerical study based on the finite element (FE) model has been conducted to analyze the stress distribution of the different thicknesses of TGV-Cu. Additionally, a TGV interconnected structure model with a polymer buffer layer is given to solve the crack problem appearing at the edge of RDL. Meanwhile, after practical verification, in comparison to the experimental results, the FE model was shown to be highly effective and accurate for predicting the evolution of stress, and several recommendations were made to alleviate stress-related reliability concerns. An improved manufacturing process flow for the TGV interconnected structure was proposed and verified as feasible to address the RDL crack issue based on the aforementioned research. It provides helpful information for the creation of highly reliable TGV connection structures.

## 1. Introduction

With the diversification of integrated circuit applications, advanced packaging technologies continue to develop toward nanometer size. It is becoming more difficult to improve performance and functionality while reducing package size and manufacturing costs. Compared with the 2D system in package (SiP), 3D interconnected packaging technology features high IO density, low power consumption, and large bandwidth, which mainly can be used in the field of the integrated passive device (IPD), high bandwidth memory (HBM) and antenna in package (AiP) [1,2].

As semiconductor technology advances, the interconnection materials change from ceramics to organic materials. In terms of dimensional and performance stability, silicon and glass are better suited for fine-pitch interconnects with high IO density than organic substrates. However, silicon as a semiconductor interconnect structure, it requires dielectric layer deposition, which raises production costs. Therefore, glass has emerged as a new generation of interconnected material of choice in recent years. As an insulating material, glass has become an attractive support material for advanced manufacturing and packaging due to its adjustable coefficient of thermal expansion (CTE), excellent surface flatness, high resistivity, and low cost. Its characteristics are specifically outlined as follows. Firstly, glass has a lower dielectric constant, allowing for lower RF loss and higher linearity [3]. Secondly, compared with other materials, glass is optically transparent and easy to control the quality. Thirdly, the high temperature and chemical resistance provide good reliability for Micro-Electro-Mechanical System (MEMS) packages. Fourth, the preparation of ultra-thin glass can be linked to MEMS for ultra-miniaturized designs. Finally, through glass via (TGV) is simple to prepare in various ways. Based on the above advantages, three-dimensional (3D) interconnect with TGV technology has wide applicability in radio frequency (RF) devices, optoelectronic systems, and multi-layer glass substrates. According to Yole, demand for glass materials is already surpassing 4 million 8-inch wafers in 2019 and is predicted to reach 9.5 million 8-inch wafers by 2025 [1,2].

Early studies on glass focused on demonstrating the effects of various copper plating processes on high-frequency transmission characteristics of TGV coplanar waveguides (CPW) and verifying the electrical properties utilizing a daisy chain arrangement. The junction temperature test also shows that the glass interposer provide thermal coupling thermal coupling between chips and can be widely used in 3D products [4]. These works are filled with TGV blind via plating. In order to obtain a high depth-to-width ratio with no void in the via, the glass surface is electroplated with an excessively thick copper overburden layer, resulting in enormous strains. Glass is prone to breaking during chemical mechanical polishing (CMP) [5]. It becomes crucial to figure out how to resolve the TGV metallization reliability issue.

Some researchers have studied 3D interconnection based on glass such as via formation and TGV metallization. Vijay et al. investigated [6,7] the TGV formation by laminating a dry polymer film onto the glass surface and using laser ablation techniques. The through vias obtained using CO_2_ laser ablation have large diameters and are surrounded by micro-cracks, which pose problems for subsequent reliability. Corning [8,9] uses a fusion method to create high-strength thin glass, preventing the introduction of flaws that would reduce strength during later grinding. However, because there is not a buffer layer, the thin glass is vulnerable to lose during manufacture. Demir et al. laminated [10] a polymer dry film onto the surface of a glass wafer that had been prepared with TGVs, and used photolithography to leave a polymer buffer layer around the vias to avoid copper delamination. However, this approach has two risk points: one is the problem of the vias offset of TGV and polymer, and the other is the residual polymer left in the via, which affects the subsequent plating process.

The purpose of this study is to improve several failure modes and pass the corresponding reliability test in the TGV interconnection structure. To that purpose, this article conducts a series of studies on the reliability of this 3D TGV interconnection structure. In Section 2, we propose the TGV interconnection structure and typical failure types and study the stress analysis of TGV-Cu. In Section 3, we focused on the effects of geometric and material parameters on TGV stress, and established a relevant finite element (FE) model to determine the stress distribution and the dangerous points. In Section 4, two improved processes are presented and are crack-free. Finally, Section 5 summarizes the key findings and observations.

## 2. TGV Interconnection Structure Packaging Design

### 2.1. 3D Interconnection Structure for TGVs

**Figure 1:**
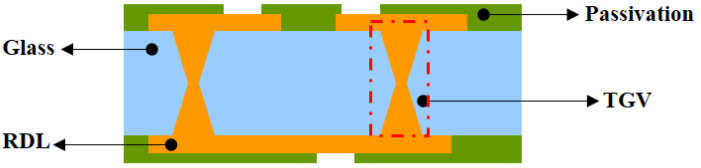
Three-dimensional interconnection structure with TGV.

Figure 1 shows the cross-sectional view of the 3D interconnection structure for TGVs. The size of the glass interposer is 10 mm × 15 mm × 230 μm. The diameter of TGV at the interior and exterior open size is 25 μm and 60 μm, respectively, with 300 μm pitch. Via array metallization will be assessed for viability. The thermomechanical performance of daisy-chain constructions will then be evaluated once they have been constructed and exposed to thermal cycling. The underlying difficulties in each fabricating process are evaluated, and the prospects for the future are examined.

**Figure 2:**
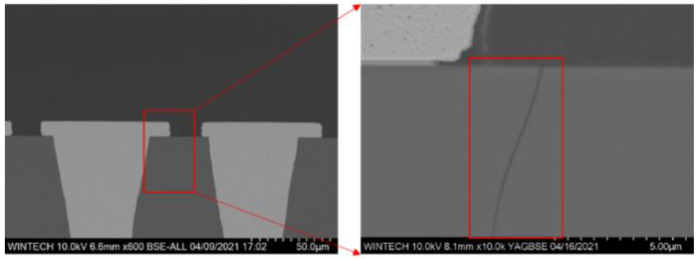
TGV package failure mode after temperature cycling.

**Figure 3:**
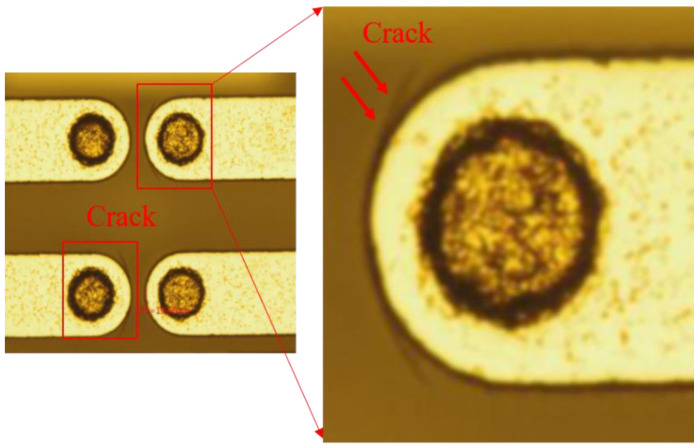
The failure mode of thermo-mechanically induced vertical cracks are observed.

Figure 2 shows the typical phenomenon of micro-crack at the edges of TGV and redistribution layer (RDL) after temperature cycling. Meanwhile, in Figure 3, another typical failure mode that occurred in our experiments vertical cracks are observed during the thermo- mechanically induced. This may be owing to the coefficient of thermal expansion (CTE) mismatch between different materials, as well as the residual stress inside the TGV–Cu structure after the high-temperature process, which leads to glass cracking. In this paper, we will analyze the mechanism of crack generation from the perspective of theory and numerical simulation and propose corresponding solutions.

### 2.2. Stress Analysis of TGV–Cu Structures

The steady-state solution of TGV delamination under heating and cooling circumstances is represented by the energy release rate (ERR) [11]. Without taking into account the elastic mismatch in the study for simplicity, the ERR for heating (ΔT>0) and cooling (ΔT<0) may be represented as:

(1) $$Cooling:G_{SS}=\frac{E{\left(\Delta \alpha \Delta T\right)}^{2}D_{a}}{4(1-v)}(1-\varphi^{2})$$

(2) $$Heating:G_{SS}=\frac{E{\left(\Delta \alpha \Delta T\right)}^{2}D_{a}(1+v)}{8\left(1-v\right)}(1-\varphi^{2})$$

where Δα, ΔT, E, v, $D_{a}$ and φ represents the thermal mismatch strain ($\Delta \alpha =\alpha_{Glass}-\alpha_{Cu}$), Young’s modulus, Poisson’s ratio, TGV diameter and the ratio of a ringed TGV’s inner-to-outer diameters. The preliminary analysis focuses on comparing the size of ERR in the two stages. The actual in-via inclination angle in this investigation is close to 85°. As a result, the effect of via inclination angle on the size of ERR values is neglected here. Through Equations (1) and (2), the ERR generated during the cooling process is greater than that of the heating process, indicating that it is more likely to lead to delamination and cracking of the TGV–Cu structure. Simultaneously, reducing the total copper percentage and the thermal mismatch strain can effectively lower the ERR.

**Figure 4:**
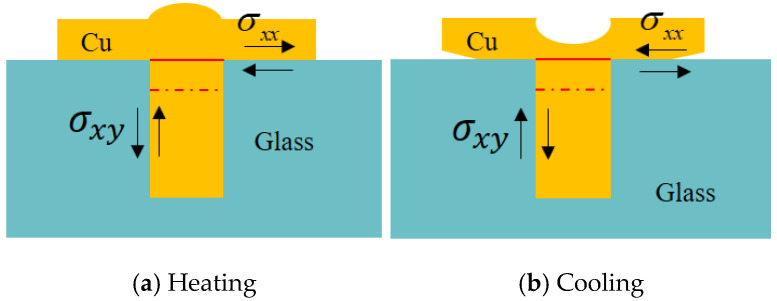
TGV delamination in the condition of opposing thermal stresses.

From Figure 4, $\sigma_{xx}$ indicates the normal stress and $\sigma_{xy}$ indicates the shear stress at the point. The different directions of shear stress indicate copper expansion out or shrinkage due to the different thermal loaded (heating or cooling). It can be seen that when the negative thermal load is loaded, it leads to delamination or crack phenomenon at the edge of TGV and RDL.

## 3. Effect of Geometric Parameters and Material on Wafer Reliability

**Figure 5:**
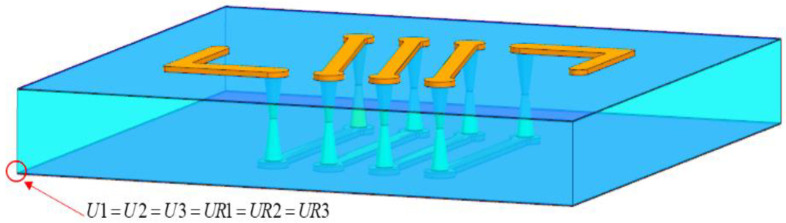
The 3D TGV structure model.

In this paper, TGV crack is primarily influenced by the thickness of the RDL, via filling, and geometric characteristics, and it is vital to fully investigate the impact of these aspects. The impact of different structural and dimensional characteristics on the stress value was investigated using the corresponding mechanical models. Figure 5 shows the model of a single interconnection structure according to the traditional process flow of copper plating design and manufacturing. In the finite element model, the package was simplified to TGV and top and bottom RDLs, and the material properties are listed in Table 1. Here, copper is given bilinear isotropic plasticity model parameters, in which the yield strength is 225 MPa and the tangential modulus is 1034 MPa. The displacement and rotation are zero at the original node (U1=U2=U3=UR1=UR2=UR3). For the curing process, the loading temperature is set to 25 °C and the curing temperature of 260 °C is set as the reference temperature.

### 3.1. Effect of Cu Plating Thickness in the Via

**Figure 6:**
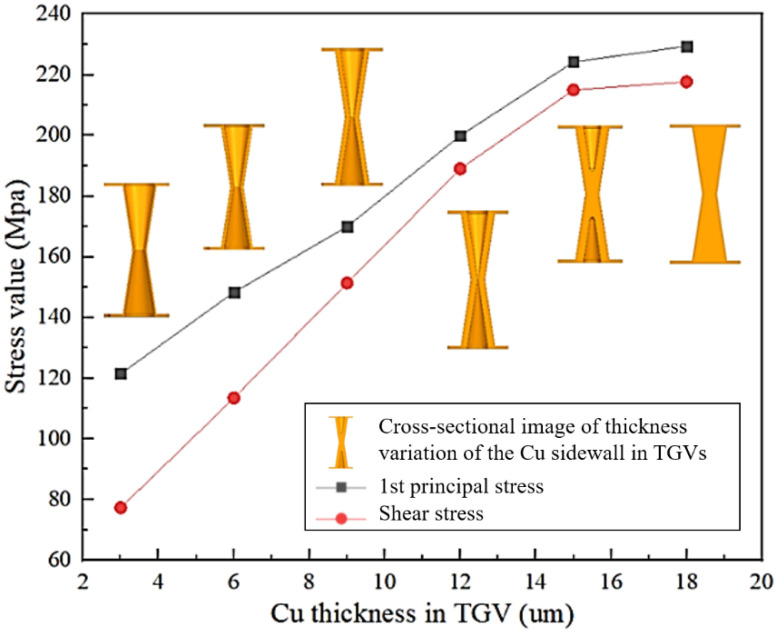
Effect of Cu thickness in TGV on stress evolution.

**Figure 7:**
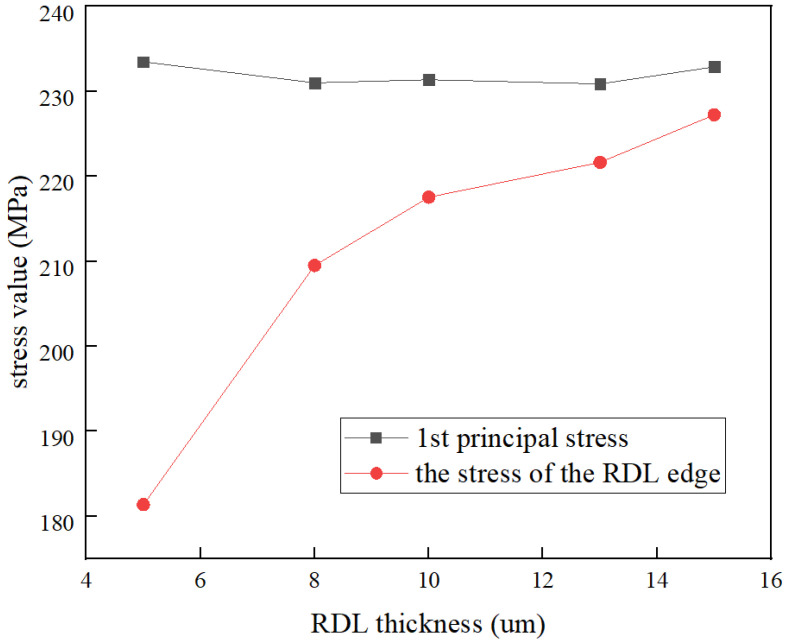
Effect of RDL thickness on the wafer on stress evolution.

**Figure 8:**
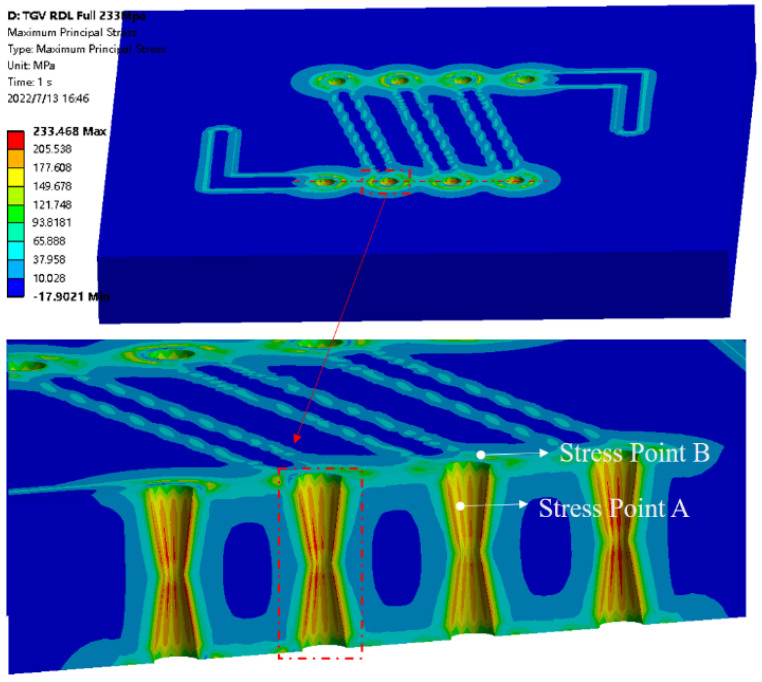
Stress distribution of the Cu-filled via on TC.

Figure 6 illustrates the values of the first principal stress and shear stress on TGV–Cu for the thickness of copper plating in the via. Here, copper thickness indicates the average thickness of copper adhering to the inner sidewalls of the via. It can be obtained from the graph that the first principal stress and shear stress are increasing with the increasing thickness of the copper layer in the via. When the thickness of the copper layer is between 15 and 20 µm, the copper on both sides of the TGV is connected together and the first principal stress growth trend slows down. Meanwhile, Figure 7 depicts the relationship between the first principal stress and RDL edge stress distribution. The stress values are increasing as the thickness of the RDL increases from 5 µm to 15 µm, which means the stress value increases by 21.62% on average. Additionally, the maximal value of shear stress is primarily focused inside the via and does not change significantly with the thickness of RDL. Figure 8 shows the dangerous points in the shear stress distribution cloud are primarily located at the edges of the RDL and inside the via, which is the main cause of crack and delamination.

### 3.2. Effect of Buffer Layers

**Figure 9:**
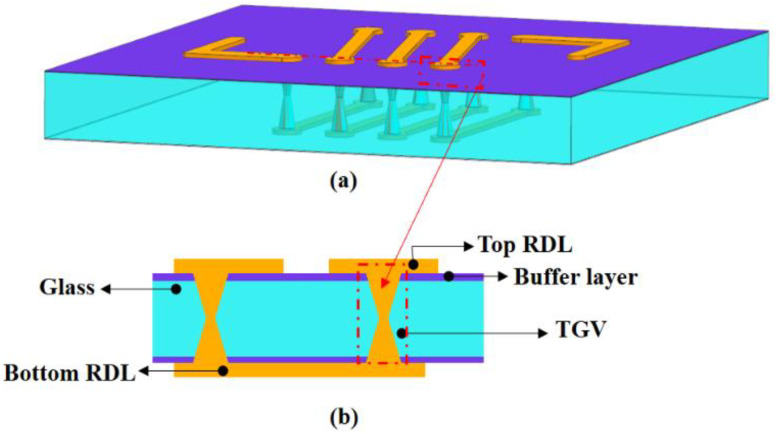
The 3D FE model. (**a**) FE model of A structure. (**b**) Cross-sectional image of A structure.

**Figure 10:**
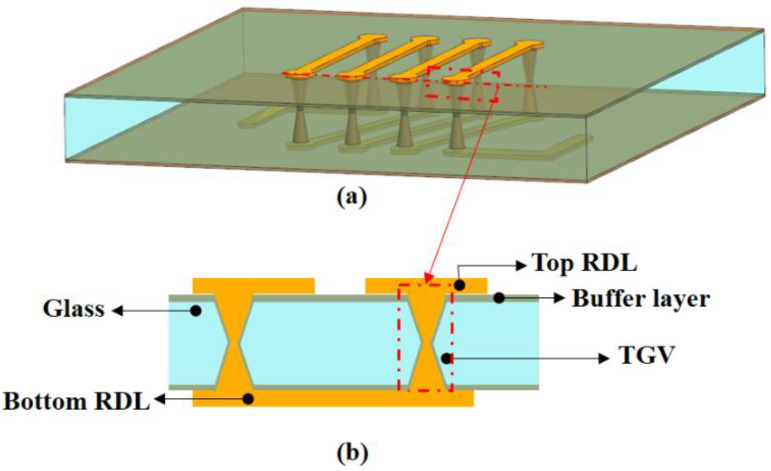
(**a**) FE model of B structure. (**b**) Cross-sectional image of B structure.

Cracks were generated by the existence of radial stress ($\sigma_{x}$) and shear stress ($\sigma_{xy}$) at the TGV–Cu interface in the preliminary theoretical and FE model investigation [12,13,14]. The elastic-plastic yield criterion was used in the analysis. When a buffer layer is added to the TGV structure, the copper expands under the temperature load. However, the buffer layer is less rigid and can be buffered over a set distance before the thermal stress reaches the glass, lowering the level as it propagates to the glass. A numerical study based on the finite element model has been conducted by designing two different buffer layer structures. The first option is to add a buffer layer to the glass surface (it was named A structure, as shown in Figure 9), whereas the second option is to add it to both the glass surface and the via (it was named B structure, as shown in Figure 10).

In order to relieve the existence of radial stress and shear stress at the TGV–Cu interface, in this paper, adding the buffer layer between the glass and the copper is a more effective method. Table 2 shows the different material properties of the buffer layer (it means the different types of polymers). Two different FE models are established for the stress evolution study in the TGVs-Cu structure. In the two models, as shown in Figure 8 and Figure 9, the packages are simplified into three components, i.e., the top RDL, buffer layer, TGV, and bottom RDL.

**Figure 11:**
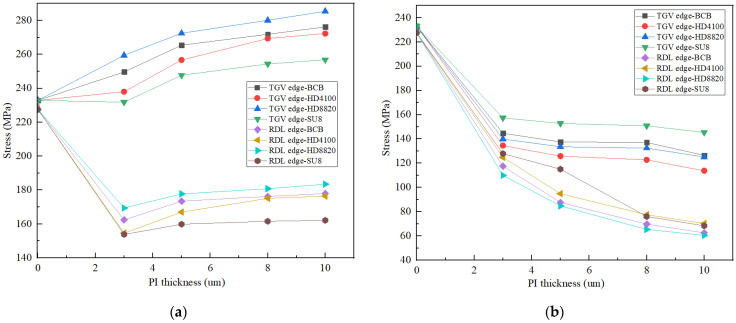
(**a**,**b**) denote the effect of different PI types and thicknesses on stresses in A and B structure, respectively.

The effect of the types and thickness of the buffer layer on the stress distribution at the steady-state cooling process (temperature conditions varied from 260 °C to 25 °C) is shown in Figure 11. It is found that the stress at the edges of the TGV was higher with the A structure than without the buffer layer, whereas they were reduced at the edges of the RDL. The possible reason for this is that the junction at the TGV edge is where polymer and copper work together on the glass, making the tension here greater. When the B structure is used, both TGV and RDL edge stress are reduced because the E and CTE of polymer are smaller than those of Cu, and polymer plays a buffering role. As the polymer thickness rises, the stress near the edge of the TGV and RDL decreases to varying degrees. In comparison to the structure without the buffer layer, the stress at the edges of the TGV and RDL was reduced by 51.15% and 69.1%, respectively, when HD4100 of 10 μm thickness was used as the buffer layer.

A thicker buffer layer can affect its electrical properties. When it is difficult to coat an ultra-thin passivation layer, consider changing the polymer to inorganic material, such as Si_3_N_4_ or SiO_2_. Table 3 shows that SiO_2_ is more effective than Si_3_N_4_. Overall, both polymers CTE and *E* will affect the stress distribution of the structure. For the selection of materials, try to choose materials with less thermal mismatch and smaller *E*. Of course, it is also necessary to consider the actual process realization. It is worth noting that when adding a buffer layer to a structure, whether it will have an impact on the RF characteristics of the entire structure needs to be analyzed depending on the specific application area.

### 3.3. Effect of Material Parameters

**Figure 12:**
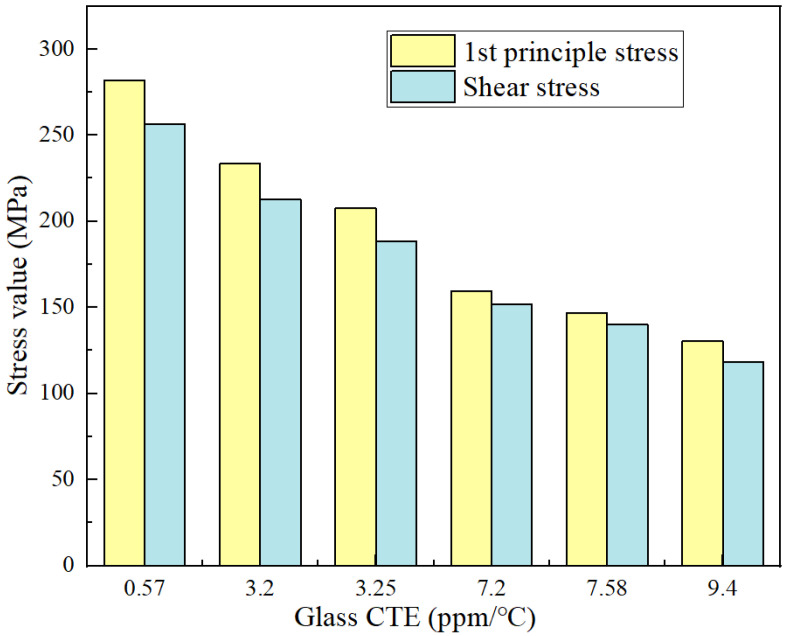
Effect of glass CTE on stress.

In the actual process, the packaging body’s stress distribution values are also affected by the material properties. Additionally, the electroplating copper layer is a common and stable process, whereas copper is an interconnection material with good performance, so, for the time being, do not consider replacing the interconnection material [15]. Conversely, glasses with different Young’s moduli and CTE are obtained by changing the metal composition in it. The results of the effect of glass with different CTE on stress are shown in Figure 12. The maximum primary and shear stresses in this structure decrease as the CTE of the glass increases, according to the trend. When the CTE of the glass was increased from 0.57 ppm/°C to 9.4 ppm/℃, both principal and shear stress in the structure were lowered by 46%. This could be due to the fact that the higher the CTE of the glass, the lower the CTE mismatch with copper (16.4 ppm/℃), and the stress problem caused by thermal mismatch is reduced. Of course, while selecting the type of glass, electrical properties, RF performance, and cost should all be taken into account. When the electrical properties are satisfied, try to choose the wafer material with the least CTE mismatch as the substrate.

## 4. Improved Process Flow and Stress Optimization for 3D Interconnection

Based on the FE analysis results, tests were carried out using the following improved processes. TGV preparation, via filling, annealing, CMP, surface RDL preparation, and passivated layer curing are the primary processes.

### 4.1. Formation of Interconnection Vias

For applications in 3D interconnection structures, high-accuracy, defect-free structures in glass may be produced using laser-induced deep etching technology. The fundamental technology that makes 3D interconnection possible is TGV. Compared with the thermal laser via formation, laser-induced denaturation can optimize through via edge defects and micro-cracks. The principle of this technology is to use ultrafast laser action on glass material, causing the laser focus area to phase change, and then the phase change area in the chemical etching process to show a different reaction rate. This area reacts more quickly with the etching solution to etch the desired via size [16].

**Figure 13:**
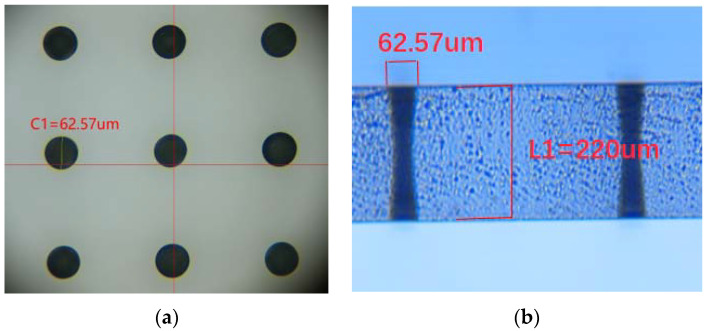
(**a**) Top view of a high-density array of TGVs. (**b**) Cross-sectional image of TGV with the specific size.

The cross-sectional image of the double trapezoidal vias following the laser-induced chemical etching procedure is shown in Figure 13. The formation of TGVs with a certain size occurs after the chemical etching process. The TGV opening size has a 62.57 µm diameter and a 220 µm thickness.

### 4.2. TGV Full Filling Preparation and RDL Formation

**Figure 14:**
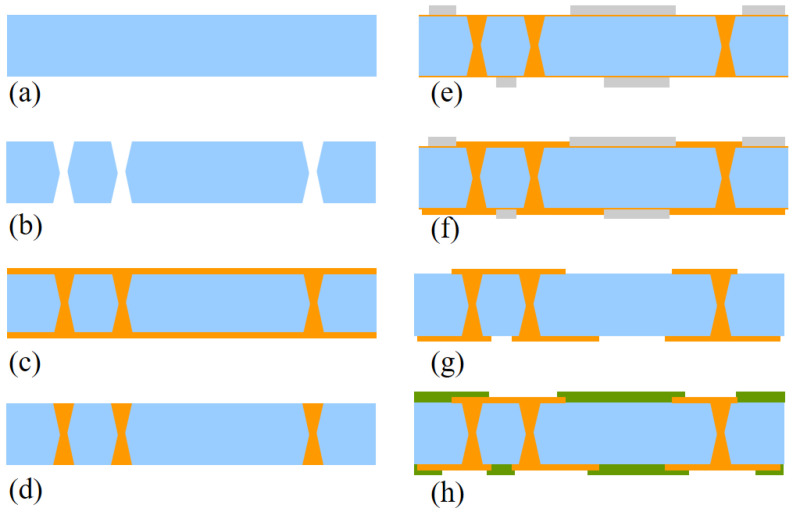
Process flow of TGV full filling. (**a**) wafer preparation, (**b**) TGV formation, (**c**) full side plating, (**d**) annealing and CMP, (**e**) PVD and photolithography, (**f**) plating RDL, (**g**) photoresist strip and Cu/Ti etch, (**h**) passivation formation.

The process flow of the TGV full-filling structure is shown in Figure 14. After TGV formation, the titanium (Ti) and copper (Cu) were formed as seed layer on the wafer surface using physical vapor deposition (PVD). Titanium and copper are 300 nm and 500 nm thick, respectively, to ensure that the seed layer is covered and continuous at the center of the via. After the electroplating process, annealing promoted the development of copper grains in the via and released stress. The copper overburden was removed by the CMP method. After secondary PVD, the removable dry film is laminated and patterned on the wafer, and the copper layer is electrochemically deposited and grown in the areas not covered by the dry film. After removing the polymer and seed layers with the wet process, the pattern is connected to the TGV to form an interconnected structure. TGV and RDL are prepared by double plating to achieve a minimal dishing value in the via. Finally, polymers with low water absorption were chosen as passivation layers to protect RDL from oxidation. Of course, the passivation layer can be opened to allow electroplating copper pillars, planting solder balls to connect with the substrate, and flip chips to the interposer packaging devices. These will be determined based on various designs.

**Figure 15:**
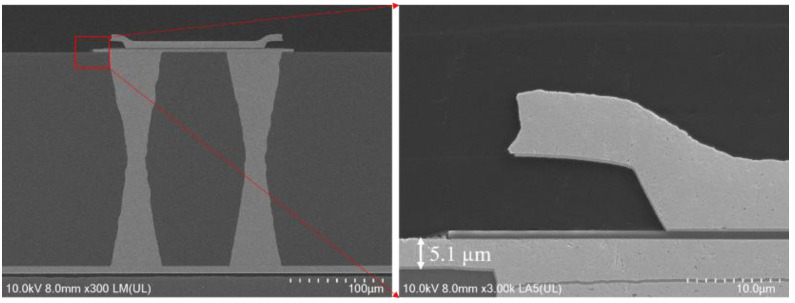
The cross sectional SEM view of TGVs with Cu full filling and RDL layer.

Reliability is greatly improved by reducing copper thickness, according to the study in Section 3 of this work. No cracks are discovered in the experiment when the thickness of the surface RDL is set to 5 µm, and the cross-sectional SEM image of TGVs is shown in Figure 15.

### 4.3. TGV Conformal Filling Formation

**Figure 16:**
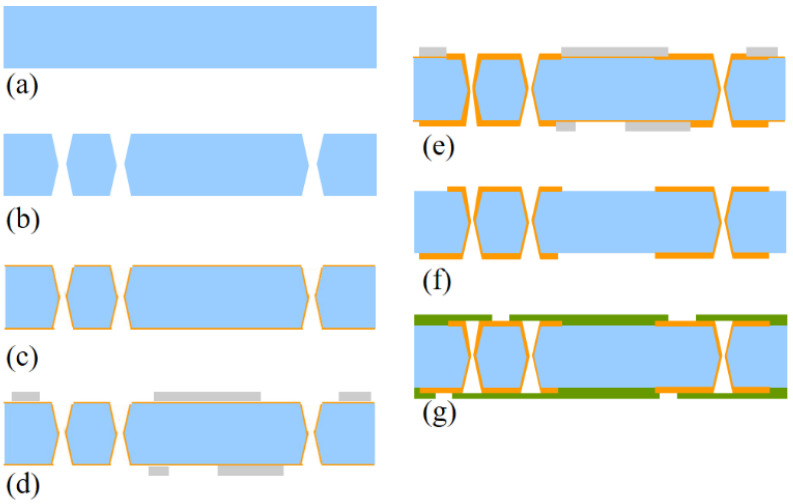
Process flow of TGV conformal filling. (**a**) Incoming glass wafer, (**b**) TGV formation, (**c**) PVD, (**d**) RDL lithography, (**e**) Cu conformal filling, (**f**) photoresist and seed layer by wet etch, (**g**) dry film lamination.

**Figure 17:**
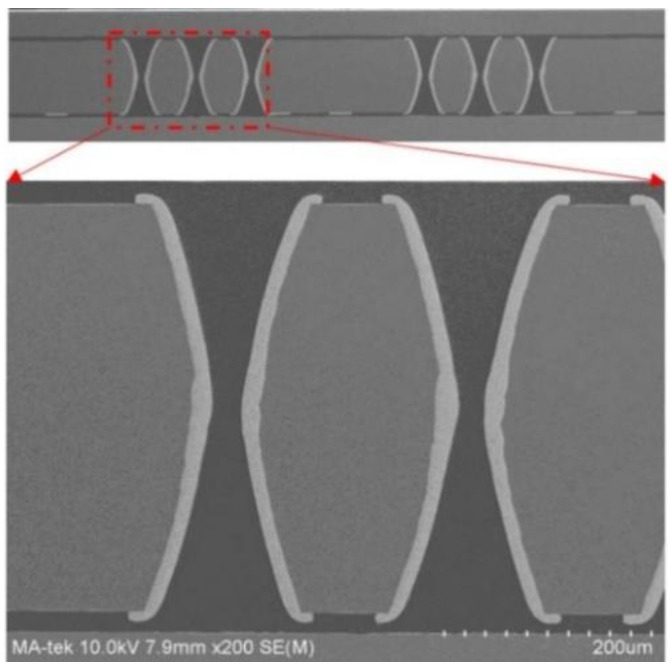
Cross-sectional SEM image of TGV metallization.

When thicker glass is required as an interconnection layer, conformal plating can be used for signal connection due to process challenges and reliability [17]. The process flow of the 3D interconnection structure is depicted in Figure 16, and the details are listed as follows: (1) we prepared a 500 μm glass wafer, and used the laser-induced deep etching technology to make TGVs. (2) physical vapor deposition (PVD), dry film lamination, and the RDL process are used to full fill Cu into TGVs and form the top and bottom metal layer. Figure 17 shows the cross-sectional SEM image of the TGV after the double plating. To ensure electrical conductivity, the plating thicknesses are all larger than 10 μm and the homogeneity is within 20%.

### 4.4. TGV Interconnection Structure Formation with a Buffer Layer

**Figure 18:**
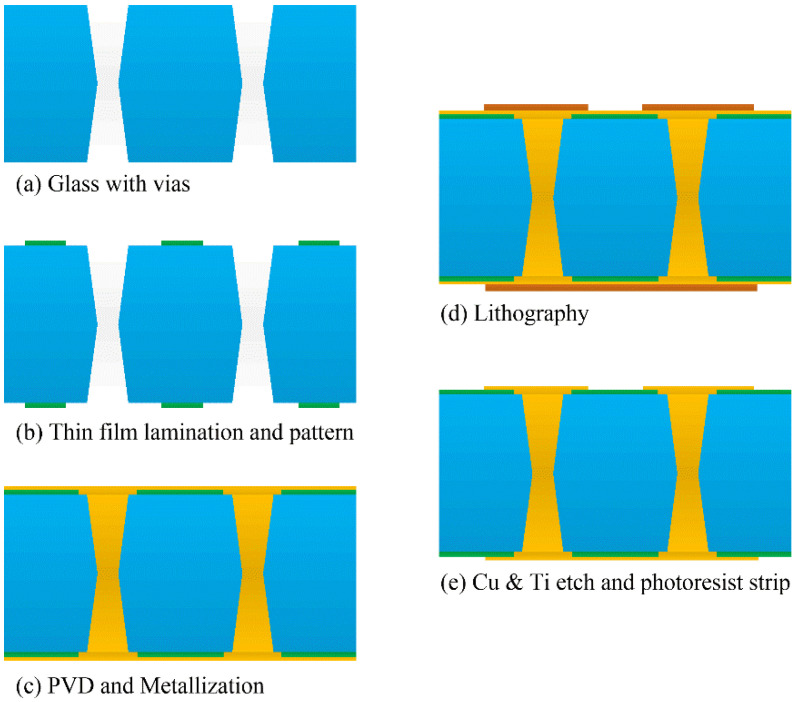
The manufacturing process flow of TGV interconnection structure by laminating a thin polymer film for a buffer layer.

In order to eliminate thermo-mechanically induced cracks in fully metalized through-glass via (TGV) substrates. The polymer film provides mechanical support and also acts as an adhesion layer between glass and the metal. This paper explores a novel approach to metalizing copper on the polymer film, which maintains the advantages of improved good metal adhesion and thermo-mechanically stresses on the edge of the TGV structure. Figure 18 shows the process flow of this approach. The process begins by laminating a thin polymer film over glass with through vias, followed by patterning the primer to open the through vias. Next, Cu metallization is created using a conventional technique, which includes seed layer deposition, copper plating, photoresist patterning, seed layer etching and photoresist stripping. Finally, yielding a structure that is known to be reliable.

## 5. Conclusions

Some issues, such as cracks appearing in the 3D-TGV interconnection structure, are explored in this work using both experimental and numerical simulations. The finite element approach is used to examine the influence of geometry and material characteristics on the TGV interconnection. Finally, some process flow and improvement measures are offered. The main conclusions were described as follows.

(1) The copper percentage in the via, the thickness of the surface RDL, and the addition of the buffer layer all affect the stress distribution in the TGV structure. For the 3D-TGV interconnect structure, the stress increases with the increase in the copper percentage inside the hole, and the maximum stress value is concentrated inside the via. As the thickness of RDL increases, the stress maximum points are mainly distributed at the edges of TGV and RDL, and this part becomes the dangerous point of failure.
(2) The CTE of different types of glass has the most significant effect on the stress in the interconnect structure, and it was found that reducing the value of thermal mismatch strain between glass and copper is one way to reduce the stress.
(3) Adding a buffer layer between the glass and RDL can significantly improve stress-related reliability issues.
(4) Reducing the surface RDL thickness or the hanging wall thickness in the via is an efficient technique to improve reliability in the actual process.

## Figures and Tables

**Table 1:** Material properties of 3D interconnection structure.

Material	Elasticity Modules (GPa)	Poisson’s Ratio	CTE (ppm/°C)	Tg (°C)
Cu	120	0.3	16.4	
Glass1	72.7	0.16	0.57	
Glass2	74.8	0.238	3.2	717
Glass3	64	0.2	3.25	525
Glass4	72.9	0.208	7.2	557
Glass5	69.3	0.212	7.58	
Glass6	71	0.2	9.4	542

**Table 2:** Material properties of 3D interconnection structure.

Material	Elasticity Modules (GPa)	Poisson’s Ratio	CTE (ppm/°C)	Tensile Strength (MPa)
HD4100	3.5	0.3	35	200
BCB4000	2.9	0.34	42	87
SU-8	4.1	0.28	50	NA
HD8820	2.3	0.25	60	170
SiO_2_	69	0.17	0.6	45
Si_3_N_4_	300	0.26	3.5	NA

**Table 3:** Statistics of stress value with SiO_2_ and Si_3_N_4_ for the structure A and B.

Items	Structure A		Structure B	
Material	SiO_2_	Si_3_N_4_	SiO_2_	Si_3_N_4_
The stress of the TGV edge (MPa)	259.92	257.63	132.64	140.82
Shear stress in the side of the via (MPa)	256.35	256.03	132.74	127.58
The stress of the RDL edge (MPa)	141.72	157.21	117.16	100.49

## Data Availability

Not applicable.
